# An unusual cause of acute anemia in an immunosuppressed patient

**DOI:** 10.1093/gastro/gov005

**Published:** 2015-02-16

**Authors:** Jamak Modaresi Esfeh, Whitney Jackson, Kianoush Ansari-Gilani, Brian Putka

**Affiliations:** ^1^Department of Gastroenterology & Hepatology, Cleveland Clinic, Cleveland, OH, USA; ^2^Department of Diagnostic Radiology, Washington University, St. Louis, MO, USA

**Keywords:** gastrointestinal bleeding, gastric mucormycosis

## Abstract

Gastrointestinal mucormycosis is an uncommon, invasive, opportunistic fungal infection with a high mortality rate, seen more commonly in immunocompromised patients. This lethal infection has a wide range of presentations, from colonization of peptic ulcers to infiltrative disease and eventually vascular invasion. Here we present a case of upper gastrointestinal bleeding in an immunocompromised patient, which was proved to be secondary to gastric involvement by mucormycosis.

## Introduction

Gastrointestinal mucormycosis is an uncommon and invasive, opportunistic fungal infection with a high mortality rate, seen more commonly in immunocompromised patients [1]. All portions of the gastrointestinal tract can become involved, with the stomach being the most common site [2]. This lethal infection has a wide range of presentations, from colonization of peptic ulcers to infiltrative disease and eventually vascular invasion. Here we present a case of upper gastrointestinal bleeding in an immunocompromised patient which was proved to be secondary to gastric involvement by mucormycosis.

## Case presentation

A 65-year-old male with type 2 diabetes, interstitial lung disease and rheumatoid arthritis—the latter being treated with chronic prednisone therapy and leflunomide—presented with periumblical abdominal pain and dark, tarry stool for the previous 24 hours. Laboratory testing demonstrated a drop in hemoglobin from 13.0 to 6.7 g/dL.

**Figure 1.**
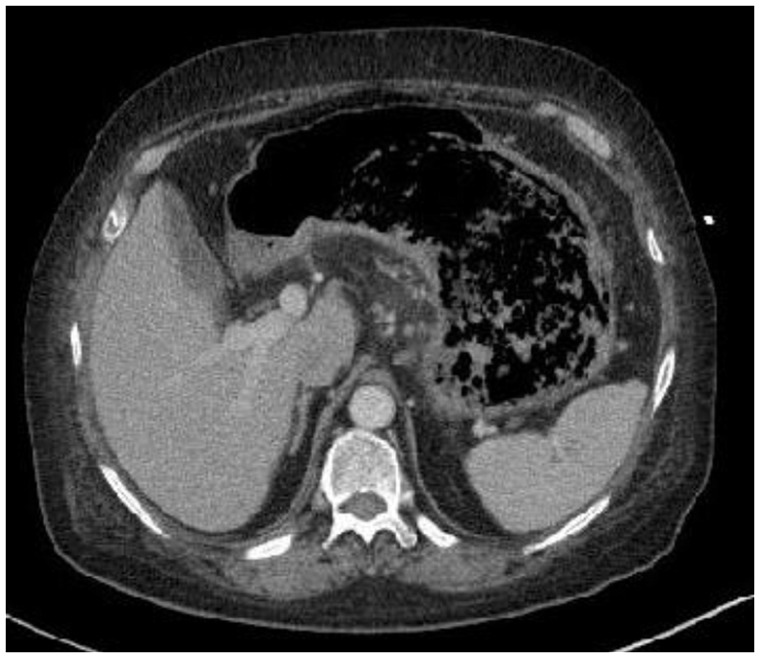
Post-contrast axial CT of the abdomen at the level of the gastric body reveals a large, loculated mass with a large amount of gas bubbles occupying most of the gastric body and fundus

**Figure 2.**
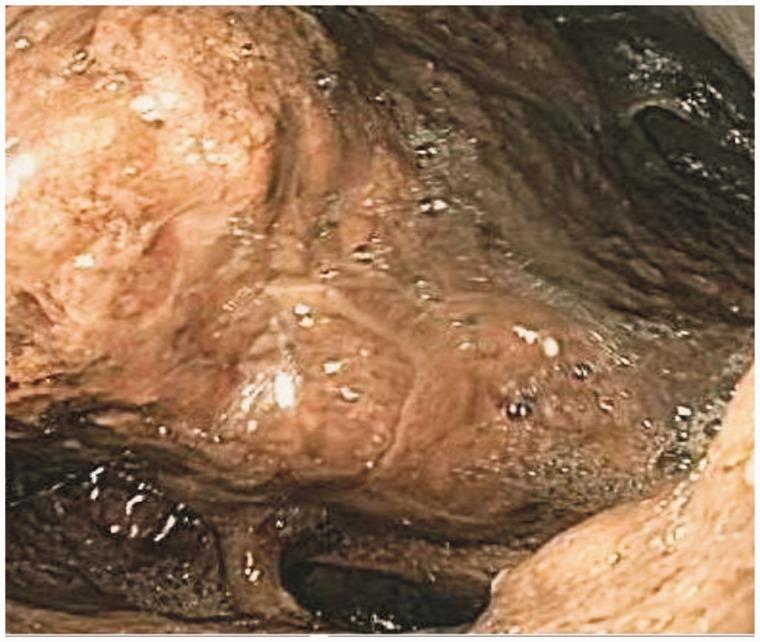
Esophago-gastroduodenoscopy shows a large fungating mass starting at the esophago-gastric junction and extending inferiorly, with obscured landmarks and dusky, ulcerated and necrotic mucosa

**Figure 3.**
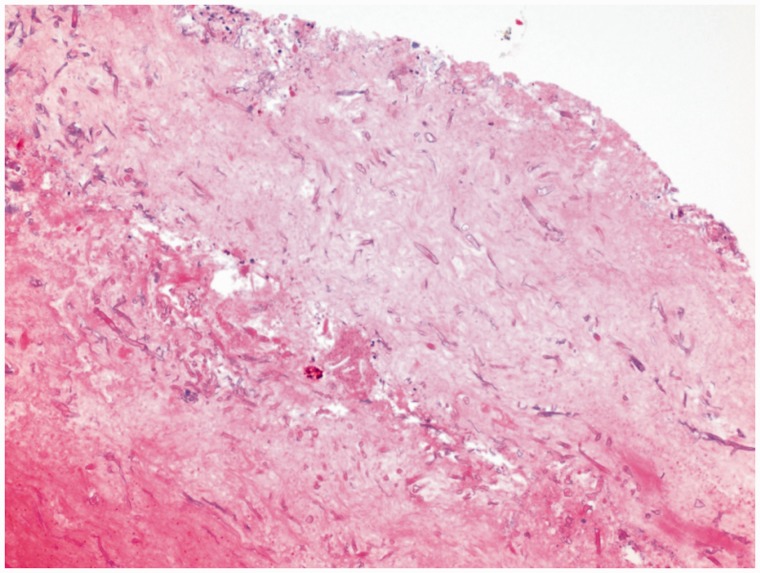
A hematoxylin & eosin section from the stomach reveals suppurative inflammation with broad, ribbon-like fungal elements infiltrating the gastric parenchyma

**Figure 4.**
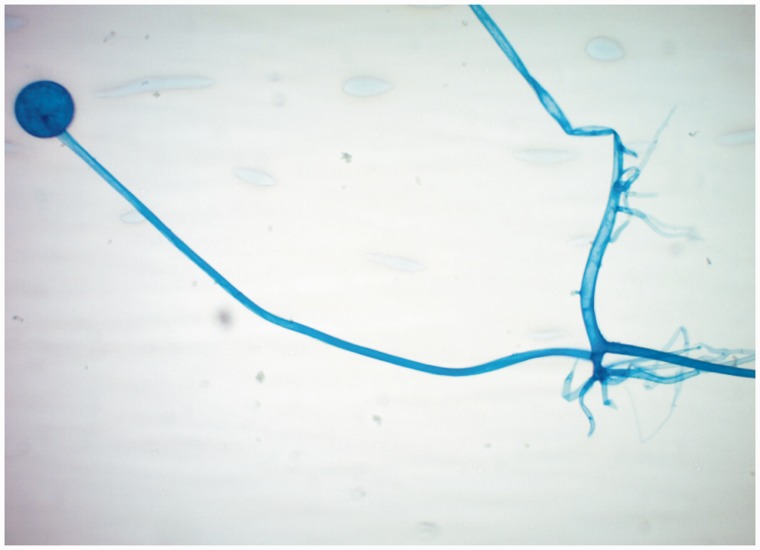
Lactophenol cotton blue adhesive tape preparation from colony shows sporangiophores directly over the sporangium, consistent with rhizopus

A large mass was found on contrast-enhanced computed tomography (CT), extending from the esophago-gastric junction to the mid-gastric body and containing extensive infiltrative gas (Figure 1). Esophago-gastroduodenoscopy revealed a large, infiltrative mass with infiltration into the surrounding gastric mucosa, characterized by dusky, necrotic and ulcerative mucosa with multiple clots (Figure 2). Biopsies were taken and revealed broad, ribbon-like fungal elements infiltrating the gastric parenchyma, compatible with mucormycosis (Figure 3). Lactophenol cotton blue adhesive tape preparation from colony, showed sporangiophores directly over the sporangium, compatible with rhizopus (Figure 4).

The patient proceeded to surgery for sub-total gastrectomy with Roux-en-Y esophagojejunostomy.

## Discussion

Gastrointestinal mucormycosis is an uncommon and invasive opportunistic fungal infection with a high mortality rate, seen more commonly in immunocompromised patients [1]. Some of the common risk factors for this infection include diabetes, inherited immunodeficiencies, immunosuppressants, solid organ- and hematopoietic stem cell transplant, malnutrition, and hematological malignancies, such as lymphoma and leukemia [2]. A meta-analysis of 929 cases revealed that the site of infection varies according to the underlying pre-disposing factor, with the paranasal sinuses being the most common site (39%, compared with 7% in the gastrointestinal tract) [3]. All portions of the gastrointestinal tract can become involved, with the stomach being the most common site [4].

This lethal infection has a wide range of presentations, from colonization of peptic ulcers to infiltrative disease and eventually vascular invasion. Invasion of the vessels by this fungus causes thrombosis, infraction and tissue necrosis, which can present as gastrointestinal bleeding [1, 4]. The mortality rate from gastrointestinal mucor infection is up to 85%, which makes early diagnosis crucial [3]. Patients should be diagnosed based on their histological findings, since the culture is positive in only 30% of surgical specimens [5]. Treatment is a combination of early surgical debridement of infected tissue, along with systemic antifungal therapy (usually parenteral amphotericin B at 1 mg/kg/day or oral posaconazole at 400 mg, given twice daily) [1, 6, 7]. Early intervention with a combined approach will give the patient a better chance of survival, up to 70% [3].

*Conflict of interest statement*: none declared.
